# Development of a nomogram for predicting myopia risk among school-age children: a case-control study

**DOI:** 10.1080/07853890.2024.2331056

**Published:** 2024-03-20

**Authors:** Jingfeng Mu, Haoxi Zhong, Mingjie Jiang, Jiantao Wang, Shaochong Zhang

**Affiliations:** Shenzhen Eye Hospital, Jinan University, Shenzhen Eye Institute, Shenzhen, China

**Keywords:** Myopia, logistic regression, influencing factor, nomogram, schoolchildren

## Abstract

**Objectives:**

To analyze the factors influencing myopia and construct a nomogram to forecast the risk of myopia among school-age children, providing a reference for identifying high-risk groups to aid prevention and control.

**Methods:**

This case-control study enrolled 3512 students from three primary schools in Shenzhen using random cluster sampling for a questionnaire survey, myopia screening and ocular biometric parameter measurement. Logistic regression was used to analyze the influencing factors of myopia, and a nomogram was constructed to forecast myopia risk. Bootstrap resampling was used to verify the practicability of the nomogram.

**Results:**

Older age (odds ratio[OR] = 1.164; 95% confidence interval [CI]: 1.111–1.219), female sex (OR = 2.405; 95% CI: 2.003–2.887), maternal myopia (OR = 1.331; 95% CI: 1.114–1.589), incorrect posture during reading and writing (OR = 1.283; 95% CI: 1.078–1.528) and axial length (OR = 7.708; 95% CI: 6.044–8.288) are risk factors for myopia, whereas an increase in corneal radius (OR = 0.036; 95% CI: 0.025–0.052) is a protective factor against myopia. The area under the receiver operating characteristic (ROC) curve of the nomogram was 0.857, and the net benefit was high when the risk threshold of the decision curve analyses (DCA) ranged from 0.20 to 1.00. The measured values were consistent with the prediction.

**Conclusion:**

The nomogram was accurate in predicting the risk of myopia among schoolchildren. This study provides a reference for screening high-risk students and for individualized myopia prevention and control.

## KEY MESSAGES

1. Older age, female sex, maternal myopia, incorrect posture during reading and writing, and prolonged axial length are risk factors for myopia among primary school students.
2. The nomogram constructed based on age, sex, maternal myopia, incorrect posture during reading and writing, axial length and corneal radius showed good predictive accuracy and practicability.
3. The nomogram constructed in this study can be used for individualized myopia risk assessment.

## Introduction

Myopia is harmful to students’ eyesight and places a heavy economic burden on society as it has become a public health problem [1], and is one of the most common health problems among children and adolescents in China [2]. Approximately half of the global population is projected to suffer from myopia by 2050 [3], and it is listed by the World Health Organization as one of the five eye diseases targeted for improvement [4]. Moreover, myopia greatly affects the lives of individuals who have it, and those with high myopia have a higher risk of eye diseases, including fundus lesions [5], macular degeneration [6], cataracts [7] and irreversible visual impairment or blindness [8]. Myopia usually occurs during childhood and adolescence, with the number of young people developing it increasing, especially in China [9]. Therefore, school-age children are a key group to target in the prevention and control of myopia.

The mechanism by which myopia occurs and develops remains unclear; however, it is generally believed that myopia is multifactorial. On the one hand, studies have shown that screen time [10] and the use of electronic devices [11,12] are significantly associated with myopia, and continuous near work is a risk factor for myopia [13]. On the other hand, the more time spent outdoors, the lower the incidence of myopia [14,15]. Furthermore, sleep duration negatively correlates with the rate of refractive error [16]. After controlling for environmental factors, parental myopia has been proven to be a risk factor for myopia [17]. Analyzing the influencing factors of myopia can help identify high-risk groups and provide evidence for early intervention. However, few studies have evaluated the effects of various factors on myopia. In addition, the myopia risk predictive models that have been constructed to date are overly complicated for quantifying myopia risk reliably, limiting their application in clinical practice [18].

Assessing disease risk can help clinicians intervene early. In this regard, nomograms have received increasing attention in the medical field in recent years as tools for disease risk assessment. Nomograms assign scores to the influencing factors of outcome events according to the degree of influence of the variables in the risk assessment model of a disease. The total score of a nomogram is obtained by adding the scores of each influencing factor. The prediction probability of an event is calculated using the function conversion relationship between the probability of an event and the total score. Finally, a graphical representation of the prediction model is presented. Nomograms can convert regression equations into visual graphs that can intuitively predict the individual risks of a disease and can be readily applied in clinical practice [19]. This study aimed to develop a nomogram for preventing myopia in child and adolescents using data on eye health, living habits, genetic factors and ocular biometric parameters.

## Methods

### Research design and participants

This case-control study was conducted in June 2022, with 3512 students from three primary schools in Shenzhen (846, 629, 612, 443, 551 and 431 students from grades 1 to 6, respectively) recruited through random cluster sampling for a questionnaire survey, myopia screening and ocular biometric parameters measurement.

Students who were residents of Shenzhen for more than 6 months and those whose parents or guardians provided informed consent for participation in the study were included. Those with ocular diseases and inability to complete the tests and investigations in the study were excluded. The sample size was estimated based on the multifactor logistic regression formula proposed by Hsieh [20]. This study was approved by the Ethics Committee of the Shenzhen Eye Hospital (No: 20201230-06), and informed consent was obtained from the participants’ legal guardians.

### Questionnaire survey

An eye health questionnaire was designed for this study. The questionnaire included questions about sex, age, body mass index (BMI), paternal (PM) or maternal myopia (MM), premature infants (PI), breastfeeding, intake of sugared beverages (SB), sweet food, fried foods (FF), fruits and vegetables, sleep duration (SD), living area per person (LAP), per capita monthly income (CMI) and posture during reading and writing (PRW). The following postures during reading and writing were judged to be correct in this study: the distances between a book and the eyes, the end of a finger and nib, the chest and the desk were 33.3 cm, 3.3 cm and a fist, respectively. The questionnaire survey was conducted twice for 100 students, with an interval of one day. The results showed that the correlation coefficients between the two measurement results of each item were >0.95, indicating that the questionnaire had good stability.

### Myopia screening

Visual screening was conducted in accordance with the Chinese Health Industry Standard (WS/T 663-2020) [21]. All participants were examined by an optometrist utilizing an electronic visual acuity chart.

The refraction test was performed in accordance with the Chinese Health Industry Standard (WS/T 663-2020) [21]. Noncycloplegic refraction was performed by an optometrist using an auto-refractor, and the spherical equivalent refraction (SER) of the participants was recorded.

Participants whose uncorrected vision acuity was <5.0 and SER was < −0.50 dioptre (D), as well as those who wore orthokeratology lens, were considered as having myopia.

### Ocular biometric parameters

Optical biometry was used to measure the ocular biometric parameters. The parameters measured included central corneal thickness (CCT), corneal radius (CR), axial length (AL) and anterior chamber depth (AD).

### Statistical analysis

R software (R Foundation for Statistical Computing, Vienna, Austria) was used for statistical analysis. The chi-square test was used to analyze the differences in the prevalence of myopia between groups, and the normality of the continuous data was analyzed using the Kolmogorov–Smirnov test. In the present study, age, BMI and ocular biometric parameters were normally distributed. Quantitative data are expressed as the mean ± standard deviation, and the correlation between quantitative data was analyzed using Pearson’s correlation. Multivariate logistic regression was performed to analyze factors influencing myopia, and OR and 95% CI were calculated. A nomogram for evaluating myopia risk was constructed using the influencing factors filtered out by multivariate logistic regression. Calibration and DCA curves were constructed to assess the discrimination, calibration and practicability of the nomogram. A ROC curve was constructed, and bootstrap resampling was repeated 100 times to internally verify the nomogram. Differences with *p* < 0.05 were considered statistically significant.

## Results

A total of 3512 school-age children, of whom 1778 had myopia, were enrolled in the present study. The SER (*r* = 0.868, *p* < 0.05) between the left and right eyes was highly correlated. The SER of the right eye was analyzed in this study. Age, BMI, sex, FM, MM and CMI significantly differed between students with and without myopia (*p* < 0.05), whereas no differences in PI, breast feeding and LAP were observed (*p* > 0.05) (Table 1).

**Table 1.** Baseline characteristics of participants (*n* = 3512).

Variables	Non-myopia (*n* = 1734)	Myopia (*n* = 1778)	Test statistic	*p*
Age, $\bar{x}\pm s$ (years)	8.5 ± 1.85	10.24 ± 2.24	*t* = −25.191	<0.001
*Sex, n (%)*				
Male	1007 (51.9)	932 (48.10)	*χ^2^*= 11.354	0.001
Female	727 (46.2)	846 (53.8)		
BMI, $\bar{x}\pm s$	17.84 ± 6.08	19.81 ± 27.11	*χ^2^*= −3.001	0.003
*PM, n (%)*				
No	1205 (51.2)	1149 (48.8)	*χ^2^*= 9.418	0.002
Yes	529 (45.7)	629 (54.3)		
*MM, n (%)*				
No	1143 (53.2)	1004 (46.8)	*χ^2^*= 32.987	<0.001
Yes	591 (43.3)	774 (56.7)		
*PI, n (%)*				
No	984 (50.4)	968 (49.6)	*χ^2^*= 1.888	0.169
Yes	750 (48.1)	810 (51.9)		
*Breast feeding, n (%)*				
Yes	919 (50.5)	900 (49.5)	*χ^2^*= 1.992	0.158
No	815 (48.1)	878 (51.9)		
*CMI, n (%)*				
≥10,000 yuan	856 (51.9)	793 (48.1)	*χ^2^*= 8.002	0.005
<10,000 yuan	878 (47.1)	985 (52.9)		
*LAP, n (%)*				
≥20 m^2^	1103 (49.7)	1115 (50.3)	*χ^2^*= 0.305	0.581
<20 m^2^	631 (48.8)	663 (51.2)		
SER, $\bar{x}\pm s$ (D)	−0.04 ± 0.94	−2.15 ± 1.59	*t* = 48.011	<0.001

BMI, body mass index; PM, paternal myopia; MM, maternal myopia; PI, premature infant; CMI, per capita monthly income; LAP, living area per person; SER, spherical equivalent refraction; D, dioptre; $\bar{x}\pm s$, mean ± standard deviation.

When the behavioural characteristics of students with and without myopia were compared, significant differences were found between groups in terms of fruit intake, SD and PRW (*p* < 0.05). However, SB, sweet food, FF and vegetable intake did not significantly differ (*p* > 0.05) (Table 2).

**Table 2.** Behaviour characteristics of participants (*n* = 3512).

Variables	Non-myopia (*n* = 1734)	Myopia (*n* = 1778)	*χ^2^*	*p*
*SB, n (%)*				
<3 time/week	287 (52.9)	256 (47.1)	3.114	0.078
≥3 time/week	1447 (49.4)	1522 (51.3)		
*Sweet food, n (%)*				
<3 time/week	87 (50.0)	87 (50.0)	0.029	0.865
≥3 time/week	1647 (49.3)	1691 (50.7)		
*FF, n (%)*				
<3 time/week	237 (52.9)	211 (47.1)	2.557	0.110
≥3 time/week	1497 (48.9)	1567 (51.1)		
*Fruit, n (%)*				
≥3 time/week	1217 (51)	1168 (49)	8.131	0.004
<3 time/week	517 (45.9)	610 (54.1)		
*Vegetable, n (%)*				
≥3 time/week	1258 (50.1)	1255 (49.9)	1.664	0.197
<3 time/week	475 (47.6)	523 (52.4)		
*SD, n (%)*				
≥9 h	801 (55.6)	640 (44.4)	37.735	<0.001
<9 h	933 (45.1)	1138 (54.9)		
*PRW, n (%)*				
Correct	723 (54.6)	601 (45.4)	23.288	<0.001
Incorrect	1011 (46.2)	1177 (53.8)		

SB, sugared beverages; FF, fried food; SD, sleep duration; PRW, posture during reading and writing.

AL (correlation coefficient [*r*] = 0.955, *p* < 0.05), CCT (*r* = 0.968, *p* < 0.05), AD (*r* = 0.942, *p* < 0.05) and CR (*r* = 0.975, *p* < 0.05) were highly correlated between the left and right eyes. Biometric parameters of the right eye were analyzed in this study. The AL, CCT and AD values in students with myopia were higher than those in students without myopia, whereas CR values were lower (*p* < 0.05) (Table 3).

**Table 3.** Biometric parameters of the eye of participants (*n* = 3512).

Variables	Non-myopia (*n* = 1734)	Myopia (*n* = 1778)	*t*	*p*
AL, mm ($\bar{x}\pm s$)	23.24 ± 0.79	24.27 ± 1.05	−33.029	<0.001
CCT, um ($\bar{x}\pm s$)	549.77 ± 32.38	551.84 ± 32.53	−1.883	0.06
AD, mm ($\bar{x}\pm s$)	3.05 ± 0.25	3.23 ± 0.26	−20.195	<0.001
CR, mm ($\bar{x}\pm s$)	7.87 ± 0.32	7.85 ± 0.30	1.910	0.028

AL, axial length; CCT, Central corneal thickness; AD, anterior chamber depth; CR, corneal radius; $\bar{x}\pm s$, mean ± standard deviation.

To construct the logistic regression model, the statistically significant variables in Tables 1–3 were considered independent variables, and myopia was the dependent variable. According to the collinearity diagnosis, the tolerance and the variance inflation factor of variables in the logistic regression model were *>*0.1 and *<*10, respectively. Furthermore, according to the principle of collinearity diagnosis [22], no collinearity among the independent variables was observed in this study (Table 4). The results showed that age, sex, MM, PRW, AL and CR are risk factors for myopia. Older age (OR = 1.164, *p* < 0.05), female sex (OR = 2.405, *p* < 0.05), having a MM (OR = 1.331, *p* < 0.05), incorrect PRW (OR = 1.283, *p* < 0.05) and AL (OR = 7.708, *p* < 0.05) are risk factors of myopia, whereas an increase in CR (OR = 0.036, *p* < 0.05) is a protective factor against myopia (Table 5).

**Table 4.** Collinear diagnosis of the independent variables (*n* = 3512).

Variables	Tolerance	Variance inflation factor
Age	0.686	1.459
Sex	0.907	1.103
MM	0.966	1.035
PRW	0.985	1.015
AL	0.515	1.941
CR	0.734	1.362

MM, maternal myopia; PRW, posture during reading and writing; AL, axial length; CR, corneal radius.

**Table 5.** Multivariate logistic regression analysis of risk factors for myopia among school-age children (*n* = 3512).

Variables	*β*	Standard error	Wald *χ^2^*	*p*	OR (95% CI)
Age	0.151	0.024	40.606	<0.001	1.164 (1.111–1.219)
Sex	0.877	0.093	88.355	<0.001	2.405 (2.003–2.887)
MM	0.286	0.090	9.972	0.002	1.331 (1.114–1.589)
PRW	0.250	0.089	7.859	0.005	1.283 (1.078–1.528)
AL	1.957	0.081	590.644	<0.001	7.708 (6.044–8.288)
CR	−3.313	0.185	322.269	<0.001	0.036 (0.025–0.052)

OR, odds rate; CI, confidence interval; MM, maternal myopia; PRW, posture during reading and writing; AL, axial length; CR, corneal radius.

**Figure 1.**
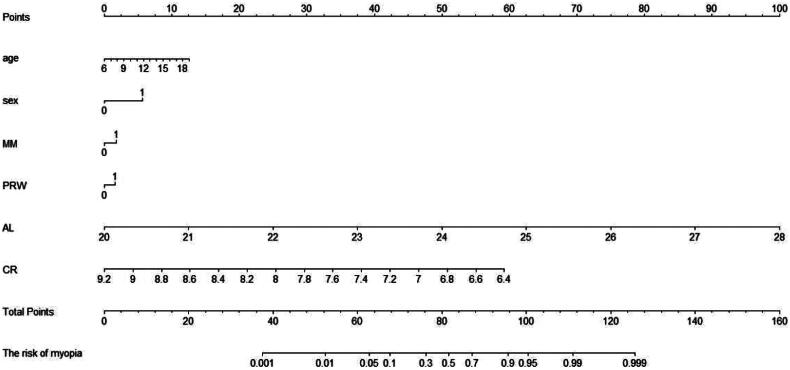
Nomogram for predicting the risk of myopia. MM, maternal myopia; PRW, posture during reading and writing; AL, axial length; CR, corneal radius. For sex, 0 = male and 1 = female; For MM, 0 = no and 1 = yes; For PRW, 0 = correct and 1 = incorrect.

Six variables with *p* < 0.05, including age, sex, presence of MM, PRW, AL and CR, were used to construct a nomogram for predicting myopia risk. The results showed that with every 1-year increase in age, the score increased by 2.5 points; for every 1-mm increase in AL, the score increased by 12.5 points; for every 0.4-mm decrease in CR, the score increased by 12.5 points; female sex scored 5 points; MM scored 2.5 points and students with incorrect PRW scored 2.5 points (Figure 1).

**Figure 2.**
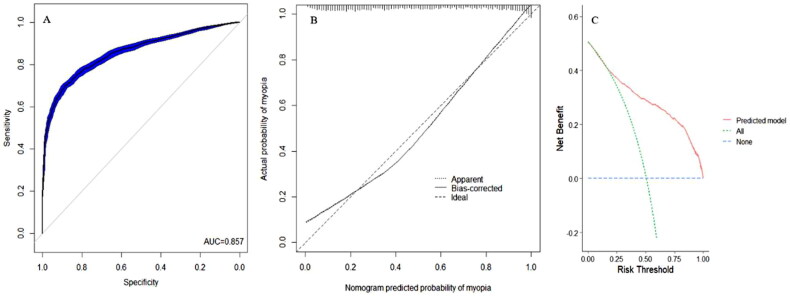
Verification of nomogram for predicting the risk of myopia. (A) ROC curve of nomogram. (B) Calibration curve of nomogram. (C) DCA curve of nomogram. ROC, receiver operating characteristic; DCA, decision curve analysis; AUC, area under the curve.

Differentiation analysis revealed that the area under the ROC curve of the nomogram was 0.857 (Figure 2A). The slope of the calibration curve was close to 1, suggesting that the model had high accuracy (Figure 2B). The DCA showed that the model had strong practicality in a wide threshold range (0.20–1.00) (Figure 2C).

## Discussion

This study explored the factors influencing myopia in primary school students and constructed a nomogram consisting of age, sex, PRW, MM, AL and CR. The nomogram developed in the present study can be used for individualized myopia risk prediction among students and provides a reference for preventing myopia. The global prevalence of myopia among school-age children has been increasing [23], reaching up to 20% in East and Southeast Asia [24]. High myopia can lead to complications, such as severe visual impairment [25,26]. Therefore, early intervention measures must be implemented to prevent the occurrence and development of myopia.

The prevalence of myopia in the present study increased with age, consistent with previous studies [27,28]. Continuous near work is related to the prevalence and progression of myopia [29], while sex has been confirmed to be associated with the occurrence of myopia [30,31]. The prevalence of myopia among female students was higher than that among male students in the present study, indicating that females are more likely to suffer from myopia than males, which is consistent with the results of studies in other countries [32]. Myopia is influenced by several factors, including environmental and genetic factors [33]. Genetic factors are often regarded as the cause of myopia [34], and evidence for the same was found in the present study – maternal myopia influenced the occurrence and progression of myopia in children.

Measurement of ocular biometric parameters is an important method in clinical research and epidemiological investigations of myopia, and the resultant observations provide indispensable objective indices for evaluating the effects of myopia prevention. The main refractive parameters include corneal curvature, lens refractive power and AL. Studies have shown that axial growth is highly correlated with changes in refractive power [35], and the main factor contributing to the occurrence and progression of myopia is axial growth. When axial growth exceeds the appropriate range for whole-eye refractive power, the overall refractive power of the eyeball develops toward myopia. The longer the axis, the more severe the degree of myopia [36]. A Danish study found that the longer the AL in school-age children, the greater the probability of myopia among students [37], which is similar to the results of the current study. Several studies have confirmed a positive correlation between CR and refractive power [36, 38]; the greater the CR, the lower the risk of developing myopia. A similar trend was observed in the present study; thus, CR is considered a protective factor against myopia.

Nomograms can visualize the results of logistic regression, intuitively predict the risk of individual diseases and can be easily popularized and applied in clinical practice [19]. Some researchers constructed nomograms for predicting myopia risk based on various factors [39], which only included demographic characteristics and ocular biometric parameters; behavioural factors were not included in those analyses. Based on demographic characteristics, genetic and behavioural factors and ocular biometric parameters, the present study developed a nomogram for forecasting and visualizing myopia risk. The nomogram has the advantage of quantifying and individualizing myopia risk assessment. The ROC, calibration and DCA curves were utilized to confirm the good practicability of the nomogram. The nomogram in the present study was constructed based on the risk factors of myopia to quantitatively evaluate myopia risk in children and adolescents. The different values of each myopia influencing factor were converted into scores in the nomogram; the higher the total score of influencing factors, the greater the myopia risk. The nomogram constructed in the current study more intuitively showed myopia risk and is easier to implement in clinical practice compared with other risk calculators.

There are some limitations to the present study. First, the noncycloplegic refraction used in this study has high accuracy in myopia screening among children and adolescents [40]. Consequently, we could not distinguish between myopia and pseudomyopia using noncycloplegic refraction and may have overestimated the prevalence of myopia [41]. Second, the relevant indices of behavioural characteristics obtained in this study were self-reported by the participants; therefore, recall biases may exist. Third, this study was a case-control study and could not confirm a causal link between influencing factors and myopia [42]; thus, we could not predict future myopia risk. Therefore, longitudinal cohort studies will be conducted to verify the association between the influencing factors and myopia risk.

## Conclusion

The constructed nomogram, which was based on age, sex, presence of MM, PRW, AL and CR showed good predictive accuracy and practicability and can be used for individualized myopia risk assessment and provides a reference for preventing myopia among school-age children.

## Data Availability

All data supporting the results reported in this study are available from the corresponding author upon request.
