# Correction

**DOI:** 10.1080/07853890.2024.2363586

**Published:** 2024-06-07

**Article title:** Development of a nomogram for predicting myopia risk among school-age children: a case-control study

**Authors:** Jingfeng Mu, Haoxi Zhong, Mingjie Jiang, Jiantao Wang and Shaochong Zhang

**Journal:** *Annals of Medicine*

**Bibliometrics:** Volume 56, Number 01, 2331056

**DOI:** https://doi.org/10.1080/07853890.2024.2331056

When the above article published online, it contained incorrect values in the following sentence. This has been corrected and republished with the accurate information.

**Incorrect sentence**

The results showed that with every 1-year increase in age, the score increased by 2.5 points; for every 1-mm increase in AL, the score increased by 12.5 points; for every 0.4-mm decrease in CR, the score increased by 12.5 points

**Correct sentence**

The results showed that with every 1-year increase in age, the score increased by 0.96 points; for every 1-mm increase in AL, the score increased by 12.5 points; for every 0.6-mm decrease in CR, the score increased by 12.5 points

